# Bug Busters: Who you gonna call? Professional development for healthcare simulation technology specialists

**DOI:** 10.1186/s41077-019-0105-x

**Published:** 2019-06-13

**Authors:** Scott Crawford, Stormy Monks, Rachel Bailey, Alaina Fernandez

**Affiliations:** 1 Texas Tech University Health Sciences Center El Paso, 210 North Rick Francis, El Paso, TX 79905 USA; 2 SimGHOSTS Research Workgroup, Las Vegas, USA

**Keywords:** Simulation operations, Healthcare simulation technology specialist, Sim tech, Professional development, CHSOS

## Abstract

SimGHOSTS introduced Bug Busters to train healthcare simulation technology specialists (HSTSs) as a way to showcase experienced HSTSs’ approaches to technology troubleshooting. This competition functions as a collaborative exchange of technology and operational solutions. It has been stated that Bug Busters is an equivalent to SimWars but for technology specialists. The competition starts with groups of individuals that function in the role of HSTSs. They are given a set of tasks that need to be completed within 10 min prior to a pending simulation scenario. The simulation room is designed with planned technical glitches, equipment that is defective, or inappropriate setup. There are multiple rounds of competition at a group level. In the final round, individual HSTSs from the advancing group complete challenges to win the competition. The number of correct tasks completed is used as the primary score determinant with time being the tie breaker. During each round, a set of distractors is introduced for a time bonus, asking technical or simulation-based questions that might be encountered in daily work. The competition demonstrates the need for HSTSs and provides an innovative way to acknowledge the complexity of their work and assess their skills. To ensure that the field of simulation continues to expand, training opportunities must be provided for all those involved in simulation. Bug Busters is one way to train and encourage “out of the box” thinking for HSTSs.

**Electronic supplementary material:**

The online version of this article (10.1186/s41077-019-0105-x) contains supplementary material, which is available to authorized users.

## Introduction

Simulation is increasingly used in healthcare simulation training of students and professionals. This has resulted in the development of simulation centers requiring personnel with technological expertise. The role of healthcare simulation technology specialists (HSTSs) has developed to meet this need [1]. These individuals are professionals with experience in audio-visual, information systems, medicine, and/or engineering primarily, but they may come from almost any background. These individuals have been referred to by many titles including sim tech, simulation technician, simulation operator, and operations specialist—among others [1–5]. For the purposes of this manuscript, the term used to describe this role is HSTS.

Personnel in this career are difficult to recruit, orient, and sustain. Educators with a healthcare background must learn to use technology that is rarely used in their profession, such as audio/visual (AV) systems and proprietary manikin interfaces, while those with a technology background must learn about healthcare and education that may not have been part of their prior training.

With the HSTS career role expanding, the need for professional training and development is growing as well. The Gathering of Healthcare Simulation Technology Specialists (SimGHOSTS) was launched in 2011 to support the HSTS career by offering educational conferences and online trainings. In addition to providing education, creating a method to showcase the broad skillset of these individuals was developed. This was accomplished through the creation of Bug Busters, a competition that has been held at simulation training events. This competition includes HSTSs who volunteer to showcase their skills related to simulation technical troubleshooting. This competition can test their anticipated job-related skills and provide a safe environment to learn technical and operation aspects of simulation planning and implementation. This includes setting up a simulation environment, reviewing technical requirements, using healthcare specific tools, and demonstrating professional interactions with learners and educators. The competition format described below was modeled on that of other conference-based simulation activities that have been used previously to train clinical care providers. SimWars and Sim Olympics are medical conference competitions that allow public display of team-based medical care [6, 7]. The purpose of this article is to describe the method and utility for training and teaching HSTSs through the SimGHOSTS Bug Busters model.

## Methods

The original planning for Bug Busters began by assessing the required knowledge and skillsets expected from the diverse aspects and job requirements of an HSTS. Planners worked to create a tiered, interactive competition to publicly showcase the expertise of the HSTS at annual conferences and stimulate discussions among participants about commonly encountered troubleshooting and workplace expectations.

A Delphi method using consensus opinion from HSTSs in the SimGHOSTS membership provided for the key components of the knowledge base and job skill requirements. These skillsets were identified to fall into eight domains: audio/visual technology, information technology, education, healthcare, management, simulation, theatrics, and research and evaluation [8]. These mirror the described domains in the 2018 version of examination blueprint for the Certified Healthcare Simulation Operations Specialist (CHSOS) certification (Table 1) [9]. These domains guide the task components of the Bug Busters competition.

**Table 1:** SimGHOSTS domains mapped to domains from CHSOS certification

SimGHOSTS domains	CHSOS domains
Audio/visual technology	Simulation technology operations
Education	Concepts in instructional design as applied to simulation
Healthcare	Concepts in healthcare as applied to simulation
Information technology	Simulation technology operations
Management	Healthcare simulation practices/principles/procedures
	Professional role: behavior and capabilities
Research and evaluation	Professional role: behavior and capabilities
	Concepts in instructional design as applied to simulation
	Healthcare simulation practices/principles/procedures
Simulation	Simulation technology operations
	Healthcare simulation practices/principles/procedures
	Concepts in instructional design as applied to simulation
Theatrics	Healthcare simulation practices/principles/procedures

This competition provides a hands-on simulation to test the skills expected of a comprehensive and adept HSTS. The scenarios mimic real-life tasks and are taken from actual workplace experience. A broad set of skills was considered when designing the tasks required for successful completion of each round of the competition.

**Fig. 1:**
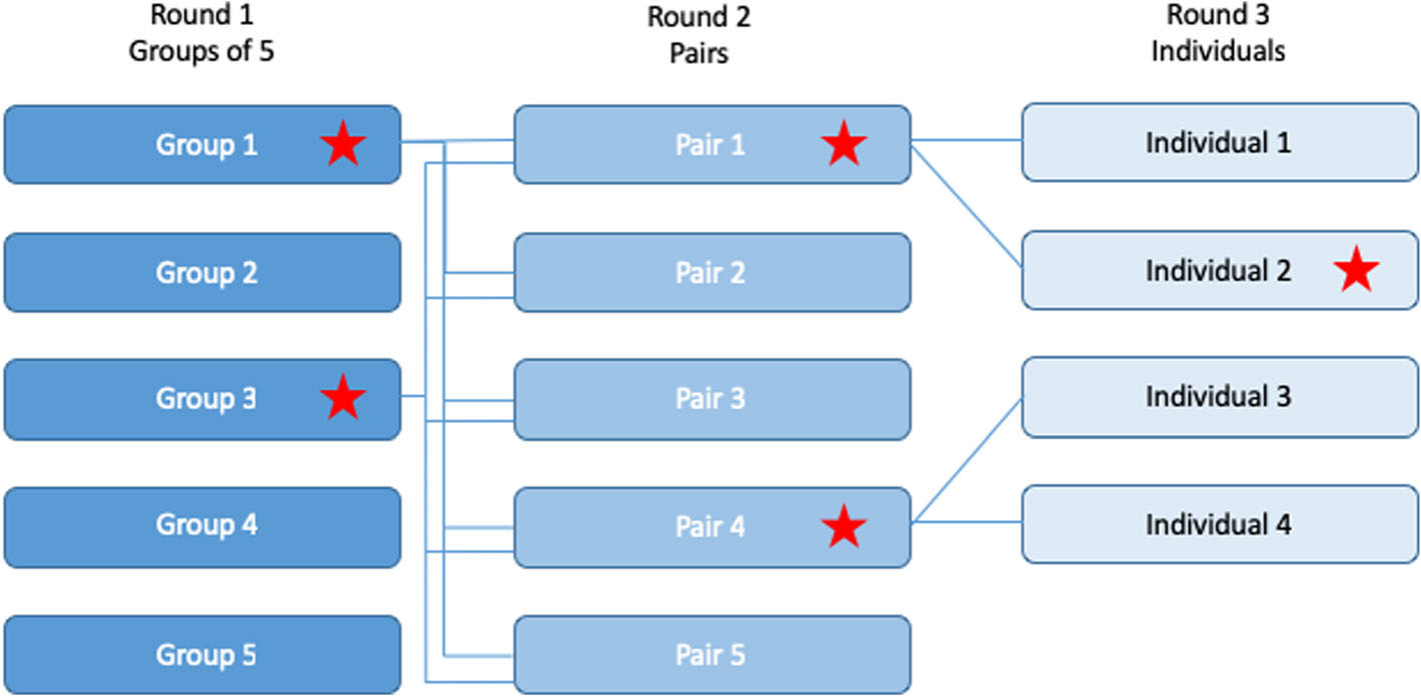
Example pairing and progression of groups through the competition

Using the round progression plan shown below (Fig. 1), the total number of groups and participants per group should be limited based on the timing available for the competition. The example shown in Fig. 1 is for 25 participants initially randomly assigned into one of five groups of five individuals, but could be configured in a similar tiered manner for almost any number of participants. The competition progresses by having the top two groups of five redistributed into five pairs for the second round of the competition, and the final round is conducted by four individuals from the top two pairs in round two (Fig. 1).

Simulation spaces around the world have a similar design; thus, the Bug Busters competition can use existing simulation spaces at host training sites. Some of the features and equipment used in the competition come from the host site. High-fidelity simulation rooms, simulation equipment manufacturers, and AV systems have similarities across regions as the same companies now distribute globally. Although some regional AV standards exist, many of the same scenario design elements can be carried between regions, especially with the help of other societies emphasizing standardized practice for the delivery of healthcare simulation-based experiences [2, 10]. Global simulation standardization allows for a reproducible competition design. This competition has been successfully implemented in multiple regions around the globe.

**Fig. 2:**
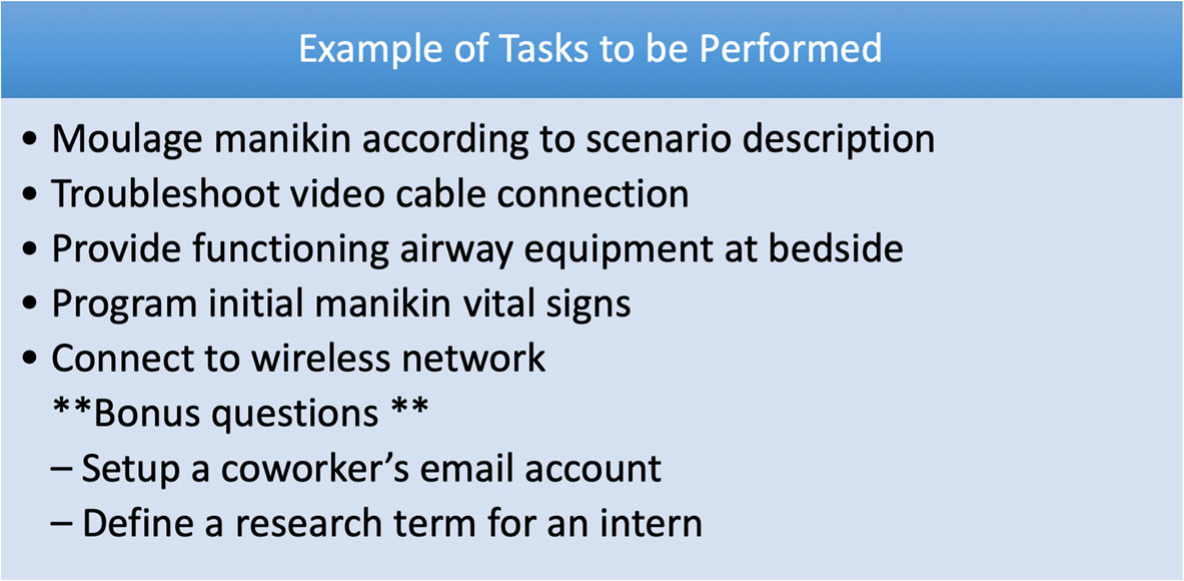
Example of tasks for a Bug Buster scenario

The simulation room used for the competition site is outfitted with technical glitches, defective equipment, and inappropriate or incomplete setup for a planned simulation. A bag of supplies required to complete the tasks, including many extraneous pieces of equipment, are provided to each group with a written summary of task expectations at the beginning of each round. The participants are instructed to prepare the simulation room based on the listed requirements of a described simulation scenario. All elements must be reviewed and tested to ensure functionality. The case description is the guide each competition participant must use to successfully finish the round. Each round is scored based on the accurate completion of each element either listed on or inferred from the summary sheet. For example, participants may be required to demonstrate mannequin setup, programming, knowledge of simulation center operations, use or interpretation of simulation or medical terminology, or troubleshoot specific audio/visual technologies. Each round is designed to have different technology, brands, and features utilized to prevent bias from individuals who may be more knowledgeable with a specific piece of hardware. Confederates may serve as distractors to impede progress or may be required for successful completion of an item on the scoring checklist. Appropriate interaction with all individuals, just as in a workplace environment, is always the expectation. Further details and scenario examples are available in Additional file 1 and Fig. 2.

### First round

In order to accommodate a large initial number of individuals with different skillsets, the first round of the competition is designed to have a set of activities that requires teamwork to be completed. This round can also require very specific knowledge that might be known by only a few individuals. This will require group interaction to share expertise for successful completion of all tasks. Items that are time intensive or involve slow technical tasks are also used during this first round as it requires learners to discuss communication strategies and planning of task execution, a critical aspect of personnel management.

### Second round

Round two focuses more on individual strengths while still having a few tasks that require the pair to work together. Items not considered to be part of general knowledge are now limited given the decrease in the number of participants for each newly formed group.

### Third round

The third and final round of the competition relies on individuals to demonstrate their skillsets in the same manner as in the previous two. However, all troubleshooting tasks should be general knowledge expected by HSTSs and should not require assistance from another individual.

### Scoring

Scoring for all rounds is based on the highest score within the allotted time. If multiple groups complete all tasks correctly, the group with the fastest completion of tasks will be declared the winner. Groups are stopped at 10 min to ensure adequate time to reset the room and maintain a schedule. During each round of the competition, a panel of three judges ensures that all items are set up uniformly between scenarios and that the timing of distractors is consistent. This panel should be consistent between scenarios, use a checklist for scoring, and also collectively decide if a task was completed successfully. The judges may be placed in the room with the participant(s), in a control room, or in another viewing area. They must be able to quickly provide redirection if participants are unsure how to interact with the simulation center environment. Appropriate and consistent moderating by the judges is imperative for case flow and ensures proper stewardship of the host center.

Even with careful attention to detail and setup, unforeseen troubleshooting tasks may be introduced if equipment that was expected to be functional does not work, just as it can occur in reality. The concept for fairness and consistency in this competition is that if it is broken in one, it must be broken in the next. If something changed between groups, it will be omitted from scoring to maintain equitable evaluation. The judges may act as a facilitator to redirect participants if something encountered is operating incorrectly or inconsistently.

During each round, one to two distractors are introduced, such as a co-worker requesting assistance with some task outside the scope of the scenario on which they are currently working. A round can also utilize directed short answer questions at the completion of the allotted time as an additional scoring option.

In order to share the experience of this activity with other conference participants, the final round can be broadcasted using the center’s AV system into the main conference hall for the other HSTSs interested in the competition to observe. Individuals participating in the competition are not allowed to observe the performance of others until they have been eliminated.

## Experience

Designing these types of scenarios can be difficult given that conferences for HSTSs are hosted at simulation centers rather than conference venues. Similar competitions such as SimWars control the presented environment by providing all aspects of the case. Using planned troubleshooting tasks that are brought for the purpose of the competition is preferred over using equipment at the center because it limits the necessity to perform a site survey looking for specific tasks to be completed at that location and limits the possibility of damaging or requiring access to secured systems. One recommendation is to keep the participants within the simulation room to limit the inherent problems from asking a technical expert to troubleshoot the wires and connections inside the control room at a host site potentially creating a real troubleshooting task for the host following the competition.

## Discussion

The competition was originally designed for entertainment purposes, but can also be used for auditioning skills during the hiring process and to assess competencies of an HSTS. SimGHOSTS has been diligent to ensure the skills that are being tested are accurate to the professional responsibilities of the HSTS. While these responsibilities may vary by region, the tasks are based on the widely accepted standards in the field of healthcare simulation. Due to the groundwork of SimGHOSTS, other facilities can take advantage of this tool when it comes to onboarding HSTSs or as a regional activity to share experience and learning between centers or across simulation regions.

It is a commonly held belief that years of experience and training can make up for the lack of a specific degree or education as qualification for a job or position, a practice that is used to grant educational equivalency for the CHSOS examination [11]. A report to the US Congress in 2014 supports this idea and described the utility of simulation to assess demonstrable job performance characteristics [12]. Just as we use simulation to assess performance of healthcare professionals, the presented model can also be used for those operating simulation-based experiences.

A survey of simulation directors conducted by SimGHOSTS, Ken Forinash, David Cohen, and Keith Littlewood from the University of Virginia in 2016 was presented at the International Meeting for Simulation in Healthcare (IMSH) in 2016. This unpublished study revealed that of 108 responses by simulation center directors, fewer than 2% found “many well-qualified applicants” for simulation specialist positions, and 81 (75%) of respondents felt most candidates were poorly-qualified for this role.

In 2016, simulation technicians in the UK were recognized by the Science Council as a professional body. This Association for Simulated Practice in Healthcare (ASPiH) recognition marks another step forward for the advancement of this specialty [13]. Specific educational programs are now offered in healthcare simulation technology, and degrees and/or certifications are becoming available [14]. Until these individuals are more prevalent in the healthcare simulation industry, education and training must be provided to develop a pool of professionals to meet the needs of simulation education.

Some simulation programs function with a limited number of staff and therefore have sought growth through the use of hybrid roles. For example, a clinical faculty may function both as educator and HSTS, leading to the requirement of training across specialties. Alinier described specific training in scenario design and implementation for both clinicians and technicians [15]. A more recent discussion in the field of healthcare simulation is whether increased expertise in the field of operations and technology will qualify individuals for advancement like the CHSE to CHSE-A and/or what these criteria may look like [16, 17]. Level of formal training and job requirements will likely help determine the differentiation between a tiered model and a hybrid role. The Bug Busters model allows the adaptation of established practices in simulation design to train and target technical and staging requirements that allow the participants to practice and demonstrate job skills, social interactions, and requirements for healthcare delivery. It is anticipated that this competition will grow and change as the needs and skills evolve in the field of healthcare simulation technology.

The need for high-quality HSTSs is clear. The Bug Busters model can be a way to assess, train, and develop both new and experienced technicians. During the hiring process, the HSTS applicant may be asked to participate in an activity to demonstrate key aspects that relate to knowledge and skill domains. Using technology- and scenario-based interview sessions has previously been described in academic medicine [18, 19]. Novice and experienced HSTSs can also benefit from training using this model to meet continuing professional development requirements beyond attending traditional lecture-based sessions [20]. Similar methods of training learners in a lively and competitive session have also been described using medically based simulation cases [7, 21]. HSTSs are integral in providing training and supporting simulation-based education. Advancing the HSTS profession will in turn lead to improved educational delivery for healthcare professionals.

## Additional file

Additional file 1: Bug Busters scenario planning—by round. (PDF 76 kb)

## Data Availability

Not applicable to this article as no datasets were generated or analyzed during the current study.

## Abbreviations

AV: Audio/visual
CHSOS: Certified Healthcare Simulation Operations Specialist
HSTS: Healthcare simulation technology specialist
IMSH: International Meeting on Simulation in Healthcare
IT: Information technology
SimGHOSTS: The Gathering of Healthcare Simulation Technology Specialists
