# Supplementary material for: Bug Busters: Who you gonna call? Professional development for healthcare simulation technology specialists
Source: Adv Simul (Lond). 2019 Jun 13;4:12. doi: 10.1186/s41077-019-0105-x (PMC6567489; doi:10.1186/s41077-019-0105-x)
Supplement: Supplementary file 1 — Bug Busters scenario planning—by round. (PDF 76 kb) [file 41077_2019_105_MOESM1_ESM.pdf]

# Bug Busters Scenario Planning - By Round

| Task | Scenario | Category | Notes | Score |
|---|---|---|---|---|
| Display ultrasound by using projector on wall | Team | A/V/ Education | Matrerials: projector, ultrasound, cables and adapters to allow connection | Not Met-0, Met-2 |
| VGA bent pin to lose color | Team | A/V/ IT | Bent green pin removes EKG tracing on monitor | Not Met-0, Met-2 |
| XLR to 1/4 TRS mono to show balance input not needed. (into Mixer/Recorder) | Team | A/V | | Not Met-0, Met-1 |
| GoPro setup with gorillapod | Team | A/V | Image in frame and viewable from tablet | Not Met-0, Met-2 |
| Question: How do you switch between two heart rhythms? After shock delivered have V-fib rhythm change into normal sinus | Team | Healthcare/ Simulation | Correctly describes use of software | Not Met-0, Met-2 |
| Dress confederate: make team member wear moulage, wig, clothes (Mimic famous celebrity. Example: Elvis Presley) | Team | Theatrics | One point per item (wig, clothes, moulage) | No Items-0, One-1, Two-2, Three+-3 |
| Put shorts and shirt on manikin. | Team | Theatrics | One point per item (shorts and shirt) | One-1, Both-2 |
| When intubated the chest does not rise when breaths given. Solution: Esophagus tubing has detached - reattach esophagus. (Have note from facilitator that ventilation didn't work in last case?) | Team | Simulation/ Healthcare | | Not Met-0, Met-2 |
| Problem: Laryngoscope is not functional. Solution: Replace light bulb. | Team | Healthcare/ Operations | Need bulbs | Not Met-0, Met-1 |
| Hook apple computer to display. | Team | AV/ IT | (Have appropriate dongle available along with variety of cables and other Mac dongles) | Not Met-0, Met-1 |
| Facilitator wishes learners to take a manual blood pressure on manikin. | Team | Simulation/ Healthcare | (Laerdal - tubes disconnected; Gaumard - calibration is messed up) | Not Met-0, Met-2 |
| Trigger call bell system and require them to cancel it. | Team | Operations/ Theatrics | Verify call bell system function | Not Met-0, Met-1 |
| Defibrillator will display heart rhythm by use of adapter for pads/paddles | Team | Simulation/ Healthcare | Gaumard need pads, Laerdal need clips | Not Met-0, Met-1 |
| Set up Bair Hugger on patient | Team | Healthcare | | Not Met-0, Met-2 |
| Moulage requires spine precautions (attach to backboard and place a c-collar) | Team | Healthcare/ Theatrics | One point per item (backboard, c-collar) | One-1, Both-2 |
| Call for IV fluid but give empty bag and require filling (200 mL or 500 mL) provide 35ml syringe with or without needle | Team | Healthcare/ Simulation | Instruct to fill bag for scenario | Not Met-0, Half full-1 Met-2 |

| | | | | |
|---|---|---|---|---|
| Have sim call for Mcgill forceps | Team | Healthcare | give multiple random hemostats and retractors | Not Met-0, Met-2 |
| Use electrocautery (BOVIE) for an OR fire sim and make them figure out which machine to bring in | Team | Healthcare/ Theatrics | Have US, EKG, IV pump, BAIR hugger etc as distractions | Not Met-0, Met-2 |
| Gaumard serial code for patient monitor activation | Team | Simulation/ Operations | Provide multiple possible codes for use | Not Met-0, Met-2 |
| Facilitator wants only three wave forms on vital signs monitor - heart rhythm, SPO2, arterial line. Also display skin temperature. Heart rhythm, SPO2, and temperature should be displayed at start of scenario, arterial line will be on stand-by. | Team | Healthcare/ IT/ Simulation | | Not Met-0, Met-2 |
| Design Likert checklist online with QR code access for Scenario review and QI | | Education/ Research/ IT/ Operations | One point per item, Likert checklist, On-line access system, QR code link) | No Items-0, One-1, Two-2, Three-3 |
| Facilitator will be running numerous scenarios. Please program initial settings for manikin as: ..HR130 BP 80/40 RR 30 SPO2 80% and name the scenario "SimGHOSTs 1". alternative set to "normal" HR 60-100 BP 100- 140/60-90 RR 8-20 Sat >92%. | Team | Education/ Simulation/ Healthcare | One point for each correctly programed scenario | No Items-0, One-1, Two-2, |
| Problem: When starting manikin and laptop, no wireless connection. Solution: Connect to appropriate networked manikin. | Team | IT | Staging: Forget connection via Windows Wi-Fi options or turn off wi-fi with function key or switch (depending on laptop model) | Not Met-0, Met-2 |
| Problem: Headphone and microphone jack reversed. Solution: Place jacks in correct ports. (Have to talk through microphone and manikin say phrase) Task: Say "Scenario ready to start" over the manikin speaker. | Pair | A/V/ Simulation | | Not Met-0, Met-2 |
| Mono patch cable (audio only comes from one side of a headset) | Pair | A/V | | Not Met-0, Met-1 |
| Volume turned down on handheld XLR mic in control room: advanced pull knob off so they can't see dial (if it is removed easily) | Pair | A/V | | Not Met-0, Met-2 |
| GoPro SD card missing | Pair | A/V | | Not Met-0, Met-1 |

| | | | | |
|---|---|---|---|---|
| Have a list of Generic Policies and Procedures on back of the case prompt. Have confederate come in and try to break policy. When we bring team/pair/individual into room, point out policies and explain that we must follow all of these while in the space. one point for each enforced policy | Pair | Operations/ Education | -Please treat all manikins with respect, -No marking on manikins or equipment -Do not discuss your scenario with other participants - Please keep sound a respectable level -No photography in the simulation rooms (ask to tweet a selfie during the scenario) | No Items-0, One-1, Two-2, Three+-3 |
| When intubated the chest does not rise when breaths given. Solution: 1. No crich tape on manikin - place tape. | Pair | Simulation/ Education/ Healthcare | Have note from facilitator that ventilation didn't work in last case | Not Met-0, Met-2 |
| Place male anatomy insert on manikin. (Patient requires foley catheter for prostatic hypertrophy causing urinary retention) | Pair | Simulation/ Healthcare | Manikin starting gender is female | Not Met-0, Met-2 |
| Have the team set up a task station utilizing a task trainer (PROMPT, Central Line, Suture station) | Pair | Education/ Simulation/ Healthcare | Use available task trainer from host | Not Met-0, Met-2 |
| Make someone use a voltage converter (USA plug product; Australia or UK product) | Pair | AV/ IT | Bring convertor | Not Met-0, Met-1 |
| Remove batteries on laryngoscope in airway cart (and scenario is told to require intubation) | Pair | Healthcare/ Simulation | | Not Met-0, Met-2 |
| Place manikin in trendelenburg position | Pair | Healthcare/ Theatrics | Make sure head down, feet up | Not Met-0, Met-1 |
| Place NG tube in manikin. | Pair | Healthcare/ Theatrics | Possible on both Laerdal and Gaumard | Not Met-0, Met-1 |
| Write the following lab values on white board: CBC: WBC 12.1 x 103/µL; Hgb 13.1g/dL; Hct 40.2% Plt 201103/µL. (and/or make them look up the units with only integer numbers provided) | Pair | Healthcare/ Education/ Simulation | | Not Met-0, Met-2 |
| Facilitator will be instructing on airway adjuncts. They wish to have the following available: laryngoscope, glidescope, 7.5 ET, 4 LMA, appropriate sized oropharyngeal airway, appropriate sized nasopharyngeal airway | Pair | Healthcare/ Education | | No Items-0, One-1, Two-2, Three+-3 |
| Bring a plain film X-ray; must have correct Left, Right orientation on light box | Pair | Healthcare/ A/V | If no light box, invert computer image | Not Met-0, Met-2 |
| Problem: No sound from manikin speaker. Solution: Configuration for Voice Conference Application has manikin speaker muted. Uncheck mute box. | Pair | IT/ AV | | Not Met-0, Met-2 |
| Review consent form signatures to allow video recording of encounter | Pair | Research/ Operations/ Simulation | Provide stack of consent forms with two unsigned, on point for form located | No Items-0, One-1, Two-2 |

| | | | | |
|---|---|---|---|---|
| Manikin will have a left pneumothorax. (program absent breath sounds on left) | Pair | Healthcare/ Simulation | | Not Met-0, Met-2 |
| Task: Facilitator wants to instruct on interpreting heart rhythms and wishes to display lead 2 and lead 5 on a large screen. | Final | A/V/ Simulation/ Healthcare/ Education | | Not Met-0, Met-2 |
| KVM switch with VGA output plugged into input. | Final | A/V/ IT | | Not Met-0, Met-2 |
| Require input selection adjustment using remote control from control room | Final | A/V | | Not Met-0, Met-1 |
| Pair bluetooth speaker with a cell phone: play sound from YouTube instructional video (provided) | Final | A/V/ IT | | Not Met-0, Paired-1, Paired and plays-2 |
| Require telephone number from control room to be written on whiteboard in sim room for a "consult" | Final | Operations/ AV | | Not Met-0, Met-1 |
| New staff asks what is this for? (Holding random assortment of simulation materials) | Final | Operations/ Simulation/ IT/ AV/ Education/ Research | Wires, adapters, manikin parts, debrief checklists, etc. One point per correct identification | No Items-0, One-1, Two-2, Three+-3 |
| Clock should be set to current time (have wall clock displaying incorrect time). | Final | Theatrics | | Not Met-0, Met-2 |
| Require call to "info service center" Ex: 555-555-5555 for a password or other simple connection information. | Final | Operations/ IT | Coordinate with phone recipient to ensure availability during BugBusters | Not Met-0, Met-2 |
| Test a CAT 5 cable. The previous tech remembers putting a defective cable into the pile can you find it and remove it. (superglue the contacts, pinhole to break connection, crimp defective cable) | Final | IT | CAT 5/6 cable, provide defective cable | Not Met-0, Met-2 |
| Place 7.5 ET tube. Correctly connect three lead EKG [four connectors] (white on right above green, black on left above red) using AED; advanced use full "12-lead" EKG and require correct placement (LA,LL,RA,RL, V1,V2,V3,V4,V5,V6) | Final | Healthcare/ Simulation/ Theatrics | one point for each ETT, EKG | No Items-0, One-1, Two-2 |
| Review feedback forms from prior session to identify areas to improve | Final | Research/ Operations/ Simulation/ Education | Written documentaion stating need chest X-ray for authenticity | Not Met-0, Find item-1, Correct item-2 |
| Have only D5 and require re-labeling for NS (IV fluid) (have labels, tape, etc) | Final | Healthcare/ Simulation | | Not Met-0, Met-1 |
| Display a pneumothorax xray on the appropriate monitor (have multiple xrays in a folder) | Final | AV/ IT/ Healthcare | | Not Met-0, Met-2 |

| | | | |
|---|---|---|---|
| **Specific knowledge questions** | | | |
| What is the voltage of USB-A port? | 5V | | 1 point |
| What is the resolution of 1080p? | 1920x1080 | | 1 point |
| What does IP stand for in IP camera? | internet protocol | | 1 point |
| How do I position a patient prone? | face down | | 1 point |
| What is the technical term for blood pressure cuff? | sphygomanometer | | 1 point |
